# The seven troubles with norm-compliant robots

**DOI:** 10.1007/s10676-023-09701-1

**Published:** 2023-04-26

**Authors:** Tom N. Coggins, Steffen Steinert

**Affiliations:** Department of Values, Technology and Innovation, Faculty of Technology, Policy and Management, Delft University of Technology, Delft, The Netherlands

**Keywords:** Robots, Social robots, Norms, Social norms, Robot ethics, Machine ethics

## Abstract

Many researchers from robotics, machine ethics, and adjacent fields seem to assume that norms represent good behavior that social robots should learn to benefit their users and society. We would like to complicate this view and present seven key troubles with norm-compliant robots: (1) norm biases, (2) paternalism (3) tyrannies of the majority, (4) pluralistic ignorance, (5) paths of least resistance, (6) outdated norms, and (7) technologically-induced norm change. Because discussions of why norm-compliant robots can be problematic are noticeably absent from the robot and machine ethics literature, this paper fills an important research gap. We argue that it is critical for researchers to take these issues into account if they wish to make norm-compliant robots.

## Introduction

Nowadays, many robots simulate what it is like to interact with another person. Researchers usually call this category of robots “social robots.” These machines express a wide range of capabilities related to communication and interaction. Nonetheless, we may classify a robot as a “social robot” if its manufacturer deliberately designed it to create the impression that it can understand and respond to human social behavior (Duffy, 2003; Dautenhahn, 2007; Nyholm, 2020, p.1–27). A well-made social robot should behave like a human plausibly would when they encounter certain social stimuli (Breazeal, 2003; Darling, 2016). They usually achieve this by mimicking context-specific behavioral patterns we expect other humans to follow during interactions (Fong, Nourbakhsh, &, Dautenhahn, 2003 Calo 2010; Coggins, 2023).

Scholars have warned that social robots may disturb their users by performing behaviors that we would likely consider inappropriate when performed by a human in the same situation (Sharkey & Sharkey, 2010; Li, van Wynseberghe, & Roeser, 2020; Licoppe & Rollet 2020). For example, suppose a hospital patient tells a robot designed for companionship serious, life-altering news concerning their health. If this robot reacted cheerfully, it could distress its user during an already emotionally demanding period of their life. Moreover, it would have failed to respond to this information with the solemnity humans generally know it deserves, potentially causing its user avoidable psychological harm. There are countless other ways social robots could upset people by missing the mark regarding appropriate social behavior. Indeed, we know that performing otherwise innocuous actions at the wrong time can elicit negative responses from others, thanks to our lived experience. For example, if we frowned after someone said they were happy, we may offend them. Likewise, if we spoke too loudly in locations that call for hushed communication (e.g., offices or libraries) we may annoy everyone within earshot. The same, we can assume, will hold for social robots.

Social scientists often call contextually specific behaviors we complete because others expect us to do so, social norms (Bicchieri. 2005; Brennan et al., 2013). In recent years, numerous researchers from robot ethics and adjacent fields have contended that we should use this sociological construct to build better robots. For instance, in their widely cited book *Moral Machines: Teaching Robots Right from Wrong*, Wendall Wallach and Colin Allen state that robots that perform social tasks “need some capacity for acquiring norms of the locale they find themselves in” (Wallach & Allen, 2008, p.108). The authors posit that robots programmed to comply with norms will recognize what actions they should and should not perform in a given social situation. Many other authors have made similar claims to Wallach and Allen over the past decade - to the extent that there is now a growing body of literature dedicated to developing norm compliant robots (Tomic, Pecora, & Saffiotti., 2018; Jackson & Williams, 2019; Carlucci et al., 2015; Malle, 2016; Malle & Scheutz, 2014; Bench-Capon & Modgil, 2017; Riaz et al., 2018).

These contributions collectively suggest that norms represent patterns of behavior that actors follow to produce positive outcomes for themselves and their peers. Thus, if we build robots that follow norms, they will generate similar results. Some authors argue that robots that observe social norms will benefit their users more than those that do not (Brinck, Balkenius, & Johansson, 2016; Bench-Capon & Modgil 2017; Jackson & Williams, 2019), whereas others have developed technical means to create robots that behave comparably to a human who understands which norms they should follow at a given time. (Malle & Scheutz, 2014; Carlucci et al., 2015; Malle, 2016; Riaz et al., 2018). The literature mentioned above generally implies or outright states that humans, and, by extension, robots, should respect norms because norms represent ethically-sound behavior. We will critique this postulate throughout this paper.

When we consult the sociological, philosophical, and political literature about norms, it becomes clear that we should not assume that following them will create good state-of-affairs. Scholars from these fields have highlighted that norms can, and often do, contribute to ethically problematic issues. Additionally, norms often represent behavioral principles people follow unreflectively until they stop following them; sometimes for unpredictable reasons. Although such observations are well-represented in the literature we mentioned at the beginning of this paragraph, ethically orientated research on social robots rarely acknowledges them. We will begin filling this research gap by interpreting relevant scholarship to contend that, in many cases, we should not rely on norms to guide our actions, nor should we uncritically assume that norm-compliant robots will be socially beneficial.

In the next section, we use pertinent sociological and philosophical research to define social norms. Afterwards, we dedicate most of the paper to outlining what we call “seven troubles with norms”. We argue that each of these “troubles” could derail efforts to make more ethical robots via norm compliance. Finally, we conclude by recommending further avenues of research and outline preliminary mitigation strategies to deal with the troubles we identified. Overall, we aim to introduce our readers to critical discussions on social norms and help researchers who wish to develop ethically-sound social robots avoid the troubles we identify by making them known and discernable. As far as we know, we are the first researchers to publish a contribution dedicated to raising concerns of these kind.

## What are norms?

In this section, we will provide a brief account of social norms to provide a theoretical basis for our subsequent discussion on their (often) problematic nature and why we should not uncritically rely on them to build better robots. We will show that norms represent patterns of behavior we observe because other people expect us to rather than actions one should interpret as good.

Sociologists generally agree that norms are internalized principles that prescribe or proscribe certain behaviors in specific contexts (Bicchieri, 2005, p.11; Bicchieri 2017, p.35). For instance, the imperatives “one should not laugh during funerals” and “one should dress in black at funerals” proscribe and prescribe a behavior, respectively Horne & Mollborn 2020a, b, p.468). Many, if not most, of our readers probably recognize and have internalized these principles. For example, anyone who has witnessed a Western European Christian funeral has seen these principles in action and knows that people who attend such ceremonies usually respect them. This example draws attention to another crucial feature of norms. Namely, we follow norms that people with whom we share group affiliations follow (Bicchieri, 2017, p.14–20). Indeed, norms are collectively internalized principles that specific groups observe (for instance, Western European Christians).

We encounter and follow norms arguably every time we interact with other people. For example, shaking someone’s hand amounts to a norm compliant action in places where this greeting is commonplace. Not only do we know we should shake someone’s hand when we greet them, but we also expect that whomever we amicably extend our hand towards will reciprocate this action (Bicchieri, 2005, p.5; Bicchieri, 2017, p.11–15)^1^. Expectations play a crucial role here. Even if someone does not like shaking hands with new acquaintances, they will likely do so anyway because they know that others expect them to act like this (Brennan et al., 2013). One person who decides they prefer to wave their hands wildly when greeting others is not following a norm but instead expressing an individual preference. However, if more people begin mimicking this behavior, and expect others to behave similarly, it may eventually become a norm^2^.

Unlike laws, religious doctrines, or codes of conduct, norms are rarely codified or formally enforced by institutions. We usually comply with norms due to interpersonal social pressure. People tend to treat others who follow the same norms as them positively. Furthermore, when someone fails to follow a norm observed by their community, they risk annoying or offending their peers, which may lead to sanctions of varying severity. Depending on how necessary a social group sees a given norm, such injunctions can range from disapproving looks to physical violence (Horne & Mollborn, 2020a, b).

Norms help humans coordinate as groups. Knowing that people will likely perform (or refrain from performing) an action because they observe similar norms to us means we can predict their behavior. We know that people probably will avoid walking close to us on city streets; because Western European urbanites tend to follow norms that dictate this (Goffman, 1966 p.151–193). Likewise, we know that our colleagues will generally ignore their phone if it rings during a meeting; as workplace norms proscribe such behavior. In both cases, someone who disregards the norms we just mentioned may disrupt an otherwise predictable social situation and make it harder for others to know what they should do next - as it has become evident that they cannot expect this person to behave as they previously expected them to.

Aside from enabling us to predict other’s behavior and vice-versa, norm-compliance marks us as members of social groups. Regardless of their size, social groups always maintain themselves through norms (Bourdieu, 2013 p.72–87). A relatively small amateur football team will have norms that its members follow. Likewise, people who work for much larger organizations, such as governmental agencies or multi-national companies, will observe norms specific to their occupation. We pick norms up chiefly via social immersion and imitation. Over time, we learn what members of groups we belong to expect from us by interacting with them and watching them interact with others. Eventually, we will likely begin behaving like someone from such a group because we want to fit in or come to respect the norms this group collectively observes. (Bourdieu, 2013; Prentice & Miller 1996). We generally do not actively decide to do this. Instead, we gradually and usually unknowingly internalize norms when integrating into a group.

Let us recap what we have said about norms so far. Once learnt, norms tell us what we should and should not do during specific social situations to ensure we can coordinate with others without generating social backlash. We usually do not learn them on purpose. Instead, we master them by intuitively imitating members of social groups. Notice that our discussion does not portrays norms as good or bad. Certainly, we may observe norms that align with our interests, preferences, or values, but this often is not the case. Indeed, people frequently follow norms that conflict with their ethical or political views. While at other times, people acquire, observe, or abandon norms for unpredictable, often arbitrary reasons. In the next section, we will evidence these claims by outlining seven troubles with norms and their ramifications for norm compliant robots.

### Seven troubles with norms

As stated in the introduction of this paper, numerous social scientists, political theorists, and philosophers (many of whom we will cite throughout this paper) have shown that humans often observe norms that they do not endorse for various reasons. Or unknowingly accept norms that do not align with their wants or needs. In this section, we will outline seven troubles with norms we identified by interpreting relevant scholarship on norms. To date, researchers have primarily used these troubles to highlight how human norm-compliance can lead to ethically questionable outcomes. We, however, will employ these insights to critically investigate norm compliant social robots.

Although we are the first researchers to produce a catalogue of this kind, our readers should treat the seven troubles listed below as a critical introduction to this topic rather than an exhaustive review of the problems surrounding norm compliancy. There are likely many more troubles with norms that we could have identified. We hope that other researchers will be inspired by what we have to say to identify further issues with norms and how they relate to social robots.

### Norm biases

We will begin by outlining likely the most straightforward way norm compliance can produce outcomes that negatively affect some individuals – what we call norm biases. As made clear in Sect. 2 of this contribution, members of groups tend to subscribe to the same norms as other members. If we do not belong to a group that observes a norm, we might not even know it exists or fail to acknowledge its significance. We are biased toward one way of doing things and sometimes act inappropriately when among people who do not share this bias. We contend that norm-compliant robots may express such biases and therefore respect one group’s norms while transgressing another’s.

Let us start with an example. Readers from Anglophone countries, who have never visited the Netherlands, are probably unaware that Dutch people commonly do not shake hands when they meet friends or acquaintances of a different gender. Instead, they kiss one another three times on alternate cheeks. Dutch people generally observe this norm, whereas British people do not and tend to greet everyone by shaking their hands. As such, someone from the United Kingdom may mistakenly assume that Dutch people observe this culturally specific norm too. If this hypothetical Briton visited the Netherlands, they could embarrass themselves (and others) by extending their hands toward someone who has leaned forward to exchange three kisses with them. This person’s cultural bias toward one way of doing things would result in them transgressing a local norm by accident.

People make mistakes like this all the time, especially in multi-cultural contexts where group-specific norms clash with one another. Although these errors are often more-or-less harmless, this is not always the case as we will show in a moment. Furthermore, we often do not realize that our actions will transgress norms we do not usually follow due to our cultural background until we have committed said transgression. In such cases, we fall prey to biases we did not know we had before our contextually inappropriate actions brought them to light.

We will now apply these insights to norm compliant robots. Suppose a company based in western Europe wishes to create a receptionist robot that greets, welcomes, and helps visitors as they enter a building. Such machines already exist (Licoppe & Rollet, 2020). If this company decided to make this robot norm compliant, they may develop a catalogue of behaviors people expect receptionists to observe, then program their robot to follow suit. The literature on norm compliant robots generally suggested that we should consult relevant stakeholders and experts when developing such a catalogue (Wallach & Allan, 2008, p.83–99). For instance, the company could ask people who work in or study the service industry to determine the norms they believe a receptionist robot should follow. Ideally, the company would subsequently create a robot that respects the norms these people identified.

If this group primarily consists of Christian or irreligious western Europeans, they will likely select norms that people with these backgrounds observe. Considering that, statistically, most western Europeans have such an identity, we can assume this will be the case. As such, the norm catalogue mentioned above almost certainly will contain biases (e.g., skew towards a culturally relative way of doing things); potentially leading to situations where one group’s norms receive preferential treatment over another’s. For instance, in western Europe, people commonly expect others to remove clothing that covers their face, such as sunglasses or scarves, when they enter a workplace. Indeed, when someone forgets to do this, others often remind them that they should. Suppose the robot receptionist upholds this norm by politely asking visitors to remove face-covering garments. While most visitors may comply with this request without hesitation, Muslim women who wear a hijab have reason to object to it; and may feel that the robot (and its owners) have disrespected them. In this case, the people tasked with developing a norm catalogue for this robot failed to account for some Muslim women’s choices and religious practices, therefore helped create a robot that enforces culturally relative norms that this group (women who wear a hijab) do not observe.

Mistakes like this are bound to happen. We often forget or fail to realize that people with different backgrounds from us do not subscribe to the norms we consider important. Therefore, efforts to catalogue the norms robots should follow in specific contexts will almost certainly express biases of this kind, potentially leading to the creation of ostensibly norm-compliant robots that effectively favor one group’s norms above another’s.

### Paternalism

We mentioned earlier that organizations that wish to create norm-compliant robots could ask a group of experts or stakeholders to select norms they believe a robot should observe. The academic literature on robots and norms contains numerous contributions that support this strategy (Wallach & Allen, 2008, p.83–99; Carlucci et al., 2015; Tomic, Pecora, & Saffiotti, 2018. In this section, we critique the notion that we should let some, pre-selected people decide which norms a robot will uphold. We argue that this strategy may produce robots that enforce norms an authority unilaterally decided others ought to follow for their own good. Political philosophers call such decision-making processes “paternalism” and warn us that they rob people of their right to make free and autonomous choices.

Let us begin by defining paternalism. In liberal democracies, individuals have the right to decide how they wish to live their lives, so long as their actions do not harm others (Mill, 1985, p.59–75). This principle stands among the most fundamental tenets of liberal thought (Feinberg, 1989; Dworkin, 2005). Our choices are ours to make and others should respect us as capable choosers (Rössler, 2005, p.1–17). Even if someone thinks we will make a bad decision, they should not prevent us from doing so (unless there are overriding moral reasons). If they did, they would stop us from expressing our right to decide freely and autonomously what is good for us. Liberal theorists call such attempts to control people’s decisions paternalism (Grill & Hanna, 2018). An example will help clarify this argument.

Both authors of this contribution, at some point in early adulthood, decided to pursue careers as academic philosophers. We knew that this choice was risky. Someone who wants to become an academic philosopher must complete several, often expensive degrees that take years to finish. Afterwards, they must compete with other highly skilled scholars to obtain a paid position at a university. These positions are rare and will not make one wealthy.

Consider the following hypothetical scenario. Suppose someone who knew these facts heard that we wanted to become philosophers just before we enrolled at our alma maters. They would have good reasons to question our decisions and may believe we should abandon our plans. They might think we ought not to bother ourselves with philosophy as we could pursue careers in less laborious, more lucrative fields. If this person prevented us from starting our philosophy degrees because they believed they were helping us, they would behave paternalistically. They would have decided what was good for us and forced us to conform to their will and values. Even if this action made us happier in the long run, this person would have nonetheless harmed us by robbing us of a decision that was ours to make – for better or for worse.

Letting a group of people decide what norms a robot should uphold, we contend, can produce paternalistic results. As mentioned earlier, researchers working on norm-compliant robots tend to assume that norms represent collective behaviors that individuals and social groups consider beneficial. As such, someone tasked with determining the norms a robot should observe will identify norms they believe one should follow. Indeed, why would they choose anything else? Such robots should help people. Therefore, one should program them to follow norms one considers beneficial. Much like the hypothetical character discussed in the previous paragraph, they will make these decisions based on what they think is good to do.

A robot designed this way will uphold norms some people unliterally decided were good. Suppose said robot encourages, suggests, or demands that its users observe a norm. In that case, it may compel them to comply with standards they did not choose for themselves – resulting in a robotically-mediated form of paternalism. Such instances of paternalism will vary in severity. For instance, many people frown upon swearing. A company could design a robot that refuses to respond to commands that contain utterances deemed obscene or profane to uphold this commonly observed norm, effectively ensuring that users watch their language during interaction. This design feature would force users to observe a norm someone else decided that they ought to endorse. Considering that swearing does not harm anyone^3^. liberal theory dictates that we can curse as much as we please. Therefore, restricting someone’s ability to do so amounts to paternalism.

This relatively innocuous example only scratches the surface of the many ways norm-compliant robots could create paternalistic outcomes. For instance, robots designed to simulate what it is like to interact with authority figures could compel people to observe norms they have the right to ignore (Calo, 2010). For instance, robots deployed in medical settings that stand in for nurses or doctors could command their users to lose weight or adopt a diet without their consent or consultation. Likewise, norm-compliant robots controlled by powerful institutional actors (e.g., governmental agencies or one’s employers) may persuade people to comply with norms they do not accept to avoid displeasing members of these organizations (Calo, 2010; Calo, 2011; Dobrosovestnova & Hannibal, 2021). We have the right to choose which norms we will observe. Thus, robots that compel us to follow norms we do not endorse will interfere with this right to make our own decision, resulting in paternalistic situations like the ones described in this section.

### Tyranny of the Majority

Letting users decide for themselves which norms a robot will follow seems like a logical solution to the issue of paternalism outlined in the previous section. If users collectively agreed upon the norms a robot will observe, this machine would assumedly produce less paternalistic results than one programmed by an external group. Ideally, every relevant stakeholder would get a say and help decide what a robot should and should not do alongside other people who will interact with this machine. Individual users would act like voters at polling booths and democratically select norms they believe a robot ought to follow.

Researchers have suggested numerous ways to accomplish this feat in recent years. For instance, a suitably adaptive robot could develop a norm catalogue in-situ via community feedback. Said robot would learn how to behave appropriately by continuously gathering relevant information from its users. Alternatively, one could survey users to develop a norm catalogue or let them program the robot themselves via software designed for this purpose (Wallach & Allan, 2008, p.99–117; Malle & Scheutz, 2014; Awad et al., 2018; Fuse, Takenouchi, & Tokumaru, 2019; Malle et al., 2020). In all three scenarios, the robot would ideally respect norms that most its users deem important. Such approaches would enable users to determine how a robot they collectively use will behave via processes comparable to democratic elections. The resulting norm-compliant robot would ideally reflect a user group’s actual wants and needs rather than those an external party paternalistically attributed to them.

We will argue that we should not assume that the approaches outlined above will produce outcomes that necessarily benefit a robot’s users. We will evidence this claim by outlining a well-documented problem associated with democratic decision-making called “the tyranny of the majority” and its societal consequences.

Philosophers have highlighted that democratic decision-making does not necessarily lead to just political or social arrangements since the late modern period. Alexis de Tocqueville, for instance, observed that democratic elections censor minority positions in his 1835 book *Democracy in America* (de Tocqueville 2010). Democracy, he explains, often legitimizes a majority’s interests while disregarding everyone else’s. Indeed, if most of a population desires a state-of-affairs and has the political power to realize this goal, anyone with opposing views will struggle to make their voices heard unless political measures exist to prevent this outcome (de Tocqueville 2010, p.402–427). The winner-takes-all nature of binary-choice referendums helps illustrate this point. For example, in 2016, 51.89% of British voters elected to withdraw from the European Union, leading to Brexit, whereas the remaining 48.11% of the electorate opposed this decision. For this slim majority to get what they wanted, a minority had to accept defeat and, henceforth, abide by political arrangements they voted against at the ballot box (Nyirkos, 2020, p.81).

Furthermore, letting a majority decide how things should be can prevent the adoption of valuable, heterodox viewpoints (Elster, 2014, p.158). Famed liberal theorist, John Stuart Mill claimed that majority rule can stifle social and political progress. Popular ideas, he explains, are often “dead dogmas” (Mill, 1985, p.75–119) that people accept as truthful because it is uncritically accepted and seldom, if ever, interrogated. Clinging to dead dogma prevents communities from changing their beliefs and adopting new ideas and practices that could improve their lot in life and society writ large. Mill posits that we must keep our minds open to minority positions to ensure that we do not overlook or dismiss potentially beneficial ideas, simply because most people do not support them. Considering that norms represent one popular way of doing things, some of them may amount to dead dogmas that, arguably, we should abandon due to their flawed nature.

Indeed, history shows that norms that enjoy the support of a majority can have devastating effects on people and communities. For example, white Americans generally endorsed racial segregation and the norms that helped uphold it throughout much of the nation’s history (Dorlin, 2022, p.97–111). Likewise, people living in ostensibly democratic nation-states typically considered women mentally ill-equipped to participate in politics until the early 20th century, partly due to norms surrounding femininity, therefore believed that women did not deserve voting rights (Dorlin, 2022. p.27–53). We know now that such practices and beliefs are harmful and unjust. However, they were once widely supported. Furthermore, if people of these eras had the opportunity to vote for or against the continuation of these practices, a majority likely would have elected to preserve them. They would have clung to dead dogma - a belief or norm that was rarely questioned and debated - which we now find appalling. In both cases, a minority had to fight life and limb against a majority position to persuade people to change their ways for the better.

We will now use the arguments presented in this section to interpret norm-compliant robots. Suppose 51 per cent of the people tasked with choosing the norms a robot will follow express that it must observe norm X, whereas 49 per cent of this group disagree. If we used a democratic strategy to choose between these two options, we would have to ignore 49 per cent of this group’s wishes, creating a robot that will behave inappropriately according to almost half of the people who helped program it. Much like Brexit, this result would legitimize a slim majority’s preferences and force everyone else to accept a state-of-affairs they do not endorse. One could imagine that organizations that wish to create democratically programmed norm-compliant robots would only accept results supported by an overwhelming majority to avoid outcomes like the one sketched above. However, such strategies can reinforce practices and ideas that are dead dogmas, that we may have good reasons to abandon.

We often prefer to behave one way because most of our peers do so. Such preferences do not represent the best way of doing things. Indeed, they often amount to dead dogma. For instance, most people in Anglophone countries shake hands when they meet someone. Is this the best way to greet a person? Considering that this norm spreads germs and forces people - many of whom may dislike physical contact - to touch one another, probably not. Nonetheless, we still cling to it because most of our peers consider it proper. There are countless other norms that most people within a community support, even though embracing another far less popular way of doing things would benefit them. As such, a robot programmed in this manner may uphold flawed norms that a majority endorses because said majority endorses them.

Additionally, as the examples of racial segregation and women’s disenfranchisement show, upholding how most people within a community say one should behave can lead to the reproduction of harmful, oppressive ideas and practices. Suppose an organization invites a group of people who hold uncontestably racist, misogynistic, or otherwise prejudiced beliefs to select the norms a robot should follow. These people may overwhelmingly select norms that help uphold their bigoted views. Although we hope that anyone who wishes to create norm-compliant robots will not do this, accepting these results would be the democratically justified way to program said robot.

### Pluralistic ignorance

In this section, we critique the notion that identifying norms a robot should follow by querying people about their norm preferences will produce data that genuinely reflect such preferences. We contend that the issues raised here apply to any method that assumes people will honestly convey their norm preferences via their words or actions. Since the early twentieth century, social scientists have noted that communities often collectively observe norms that many, if not most, of their members privately do not endorse (Katz & Allport, 1931). These individuals mistakenly believe that their peers generally support a norm, even though many of them also dislike it. As such, they do not reveal their views because they fear no one else agrees with them.

Social scientists call this phenomenon “pluralistic ignorance” and highlight that it is difficult to identify whether a community is subject to it, because individuals are hesitant to express their opinions as they believe others will judge them negatively for doing so (O’Gorman, 1986). We argue that accepting community members stated norm preferences as accurate fails to acknowledge the possibility that they may have expressed such opinions due to pluralistic ignorance. And a robot that relies on data derived from expressed preferences of this kind will observe norms that many community members wish their community would abandon.

People subject to pluralistic ignorance behave like the fearful subjects in Hans Christian Anderson’s fable The Emperor’s New Clothes (Miller & McFarland, 1987). In this story, the titular emperor claims that he has purchased magnificent new robes from two tailors who have tricked him into wearing nothing at all. After he appears naked before his subjects, they play along with this ruse. They assume everyone else is telling the truth, and thus do not speak up to avoid stepping out of line. Mistakenly believing that everyone else within a community agrees that one should observe a norm produces similar effects. If group members unanimously say or behave as though they endorse a norm when many of them do not, individuals who hold this opinion will “act similarly to others but assume their perceptions must be different” (Miller & McFarland, 1987). When these individuals - who may constitute the majority of a group - keep their opinions to themselves, they inadvertently contribute to the continuous observation of an unpopular norm. Furthermore, being the first person to question a norm everyone else appears to endorse is risky, as doing so may lead to embarrassment, scorn, or punishment. Hence, potential dissenters often remain silent and continue to believe their views are atypical rather than broadly supported.

Let us look at some examples from the sociological literature on pluralistic ignorance. While studying college campus students’ attitudes towards binge drinking, Deborah Prentice and Dale Miller (Prentice & Miller, 1996) discovered that many of their respondents believed that their aversion to drinking unhealthy amounts of alcohol was unique to them – even though a significant number of their peers reported that they also disliked this practice. Indeed, Prentice and Miller claim that their respondents chiefly participated in binge drinking because they felt that abstaining from this widespread practice would alienate them from their friends and classmates, whom they mistakenly assumed were committed to upholding this norm (Prentice & Miller, 1996).

Other sociologists have shown that pluralistic ignorance can help maintain political and social practices that cause grave harm. For instance, in the 1970s Hubert J. O’Gorman found that white Americans tended to overestimate other white people’s support for ‘strict racial segregation’ in neighborhoods, even though this was a minority position (O’Gorman, 1979). By keeping their views to themselves, these people allowed a practice they collectively (albeit unknowingly) agreed was unjust to persist unchallenged (O’Gorman, 1979). Likewise, Cristina Bicchieri claims that some parents continue to discipline their children with physical violence because of pluralistic ignorance. She explains that parents from communities that appear to endorse corporeal publishment directed at minors often overwhelmingly disagree with this practice. However, they continue to beat their children because they fear their peers will judge them as “weak or uncaring parents” if they do not respect this unpopular norm (Bicchieri, 2017, p.42).

We contend that pluralistic ignorance will negatively affect the development of norm-compliant robots for three reasons. First and foremost, anyone who helps decide which norms a robot will follow may express views influenced by pluralistic ignorance. Suppose an organization invites people from a given community to develop a norm catalogue for a robot. 80 per cent of them communicate a preference for norm X. However, many of them feigned this preference due to pluralistic ignorance. They sensed that everyone else endorses norm X and that they will be considered abnormal for disliking it; therefore, they did not make their opinions known to conform to their peers’ assumed beliefs. Once completed, this catalogue would contain data that does not reflect this community’s preferences and using it to program a norm-compliant robot would produce a machine that observes norms many respondents privately dislike.

The same holds for robots that learn in situ by observing group members. A robot that develops a norm repertoire by interacting with and observing users may inadvertently learn norms that many users do not endorse but follow, nonetheless. A norm-compliant robot that relies on people’s norm endorsement due to pluralistic ignorance will acquire inaccurate information that does not reflect people’s beliefs.

Second, a robot programmed this way may effectively serve as false evidence of a disliked norm’s popularity. By upholding an unpopular norm, the robot may further communicate to dissenters that their views are atypical even though this is not the case. And finally, this could make dissenters feel alienated from their community. They may continue to mistakenly believe that their views do not align with their peers’ when, in fact, they do. If they knew that other people shared their dislike of a norm, they may feel more kinship towards their community and know that their peers value the same things as they do. A robot that observes unpopular norms upheld by pluralistic ignorance may prevent this from happening by dissuading dissenters from communicating with one another. In a nutshell, a norm-complaint robot that helps preserve pluralistic ignorance will make it harder for a community to abandon a norm that many of them do not endorse and wish abandon.

### Paths of least resistance

When a community acknowledges that they should stop observing a norm because it does not align with their shared interests, they may have trouble abandoning it. As stated in Sect. 2, we tend to follow norms reflexively. We usually do not think about them, and deviating from them takes effort. As such, it is often easier to continue observing a norm even when we do not endorse it. In his 1997 book, *the Gender Knot: Unravelling our Patriarchal Legacy*, sociologist Alan G. Johnson introduces a useful metaphor to explain this phenomenon he calls “paths of least resistance” (Johnson, 2014, p.30–31).

Johnson compares norms that we want to abandon to well-trodden paths that we know will lead us to a desired destination. If we wish to get from A to B while traveling through a familiar city, we will almost certainly choose the quickest and most efficient route we know (Johnson, 2014, p.30–31.). Although this choice may save us time and energy, it does not necessarily represent the best available option. We follow this tried-and-tested path and to not go through the hassle of looking for another, possibly better route. If we discover that this “path of least resistance” has flaws, we may continue to walk down it because we find it easier to repeat past actions than deviate from them. We can liken norms we want to abandon to paths of least resistance. When we know or suspect that a norm has flaws, we may continue to observe it, as it is familiar, and finding another way of doing things takes work. In this section, we contend that norm-compliant robots may inadvertently reinforce “paths of least resistance” or disturb their users by deviating from them.

We will expound on the ideas sketched above by examining how norm compliance contributes to workplace gender discrimination. Many institutions now publicly condemn norms that prevent women from obtaining the same opportunities as men at work. Whereas it was once expected, if not required, for women to conform to gendered norms which limited their range of behaviors to those associated with femininity, ideally, they can now pursue careers without having to deal with these restrictive and unfair expectations (Hochschild & Machung, 1989, p.1–22). However, women still face difficulties when they do not adhere to norms that have historically hindered their ability to access the same opportunities at work as men (Hochschild & Machung, 1989; Babcock et al., 2022, p.1–95).

Research consistently shows that people expect leaders to observe norms concerning assertiveness, dominance, and agency (Eagly & Karau, 2002). In contrast, people generally expect women to conform to norms that convey communality, deference, and compassion (Hochschild, 2012, p.162–185; Eagly & Karau 2002; Zheng et al., 2018). When women behave as people expect leaders to behave, they transgress gender norms - thus may provoke social backlash. Female leaders often face issues at work that their male counterparts rarely encounter, partly because people generally do not expect men to observe norms that conflict with those associated with leadership (Hentschel et al., 2018). Women who observe the same norms as male leaders often receive worse evaluations from their peers and superiors, who may see them as underserving of institutional rewards such as praise, pay raises, or promotions, for this reason (Babcock et al., 2022, p.95–119) Considering that individuals and institutions often express their commitment to equality in the workplace, why do norms like those sketched above continue to unfairly affect women?

Suppose that most people working at a company believe that treating male and female leaders differently is wrong. They recognize that archaic and unfair gender norms should not influence how they see or interact with their female colleagues. Yet their collective actions do not reflect this stated belief. Women continue to experience gender-based discrimination when they observe norms associated with leadership.Although almost everyone at this company concurs that they should not ignore, unduly question, or openly disagree with commands given by women in leadership positions, many staff members continue to do so. They may neglect to listen carefully to their female colleagues when they ask them to do something. Or speak disparagingly about them once they are out of earshot. Such behaviors persist despite the company’s commitment to fairness.

We could interpret such enmity towards female leaders as the result of a path of least resistance. Unfair gender norms were the normal way of doing things for a long time. Therefore, abandoning them takes work. Many staff members probably do not realize that their behavior does not align with their shared values because they reflexively observe gender norms previously seen as acceptable. Recognizing that we have adhered to a norm that does not represent our values requires us to reflect upon our actions and take active steps to unlearn pre-learned behaviors we usually observe without much thought. Even someone dedicated to changing theirs and others’ behavior to ensure female leaders receive fair treatment may fail to act on this belief because doing so means deviating from a path of least resistance (Johnson, 2014, p.32–33, p,227–247). Challenging someone who appears to have disrespected their female colleague or supervisor could rock the boat – so to speak. They might react negatively to such criticism and feel that whoever leveled it has made it harder for them to do their jobs. As such, it may seem, and quite possibly be, easier to continue to follow this path of least resistance, as deviating from it could annoy, upset, or anger people who have not yet recognized that they should abandon a norm if they wish to treat everyone fairly at work.

Suppose an organization invites people working at a company like the one sketched above to select norms a robot should follow. Although most people believe that they should abandon gender norms that prevent women from accessing the same opportunities as men at work, they observe them without realizing it. They may inadvertently suggest that this robot should observe norms that represent their collective reluctance to stray from this “path of least resistance” and therefore do not align with their values. For instance, research shows that people tend to interpret male-coded voices as more authoritative than female-coded ones and express that they should listen more attentively to information conveyed by the former. (Nass & Moon, 2000) Thus, these staff members may recommend that a norm-compliant robot that issues commands should sound masculine, whereas one that performs communally focused tasks should sound feminine. These robots would reinforce the idea that men are better leaders than women, effectively communicating that people at this workplace endorse unfair gender norms that they collectively want to abandon.

Alternatively, they may recommend that a robot strays from this path of least resistance, perhaps by programming it to issue commands with a female-coded voice or somehow remind staff members that they should treat men and women leaders with equal respect. As stated earlier, however, deviating from paths of least resistance can and often does generate social tension. Someone who does not recognize that their actions do not match their values may respond negatively to this robot. For instance, they may dislike receiving commands from a feminine-sounding robot and complain about it without realizing that such behavior conflicts with their commitment to fairness in the workplace. Or find a robot that suggests that they should reflect upon their actions annoying or distracting.

We contend that paths of least resistance will hinder efforts to make norm-compliant robots in other contexts too. Communities regularly express commitments to values they do not uphold via their collective behavior. Although we may wish to abandon one way of doing things, achieving this aim takes effort and often disrupts pre-established, previously acceptable group dynamics that many people do not realize contribute to outcomes they do not endorse. Therefore, a norm-compliant robot’s behavior may conflict with its users’ values because it observes norms they should, but have yet to, abandon if they wish to honor their ethical or political commitments. Or unsettle users by suggesting that their actions do not reflect how they say people should behave.

### Outdated norms

We contend that efforts to create norm-compliant robots must consider that norms can and often do become outdated. As suggested throughout this paper, norms are not fixed rules that individuals learn, internalize, and observe forever. Indeed, communities regularly discard them with little warning. In some instances, communities abandon norms because they realize that said norms are (morally) problematic - as illustrated by the examples we gave earlier concerning gender inequality and racial segregation. While at other times, communities stop observing once-prevalent norms as though they have gone out of fashion. As such, a norm-compliant robot may eventually begin to act inappropriately because its users have since abandoned the norms this machine continues to follow.

When we sense that other people of our community have stopped observing a norm, we may begin to do so too. As more and more of our peers follow suit, this norm will become less prevalent and may eventually fade away altogether. Although this rarely happens overnight, norms can rapidly become outdated when a community collectively recognizes that a previous way of doing things cannot continue because it produces undesirable outcomes. Let us examine a topical example to explain the ideas outlined above. The COVID-19 crisis profoundly affected how we interact with one another, prompting us to abandon norms that we have arguably observed for centuries. Soon after the crisis began, medical organizations and governmental agencies recommended that we stop following norms that increased the spread of COVID-19. For instance, many, if not most, people in Europe acknowledged they should not shake hands when greeting someone or stand within 2 m of another person during interactions. These once-prevalent norms became outdated within the span of a few weeks. As shown by the example sketched above, we only knew we should avoid shaking hands once we learnt that this norm endangered people during the COVID-19 pandemic. We abandoned this norm because unforeseeable circumstances demanded it.

It is difficult to predict when a community will stop observing a norm mainly because it is hard to pinpoint indicators for norm abandonment and sometimes norm abandonment resembles norm transgressions. For instance, dress code norms have changed significantly over the past twenty years. Whereas we once expected white collar workers to wear formal attire, nowadays far fewer people observe this norm. Famous leaders such as Steve Jobs and Mark Zuckerberg arguably contributed to this shift by publicly appearing at work dressed in casualwear, thus signaling to others that they may do the same. Some sociologists call people who transgress norms and thus communicate to others that they may ignore them as well “norm entrepreneurs” (Bicchieri & McNally, 2018; Sunstein, 2018). If an office worker dressed like Jobs or Zuckerberg twenty years ago, a contemporary observer would likely believe that they have transgressed a norm and misunderstood or willfully ignored what people expect from them. In hindsight, however, they were among the first people to abandon this norm. As transgressions usually do not automatically lead to norm abandonment, it is safer to assume that apparent transgressions *are really transgressions* rather than actions indicating that a community will soon stop observing a norm.

We posit that outdated norms will be a challenge for the development of norm-compliant robots for the following reasons. First and foremost, communities usually abandon norms without clearly signaling that they will do so beforehand. For example, we did not know that people would stop shaking hands during the COVID-19 crisis before the fact. Likewise, we could not have predicted that certain workplace dress norms would effectively disappear or change. As such, a robot programmed to follow contemporarily prevalent norms may sooner or later perform actions that its users consider socially inept or even harmful. Knowing when this will happen would require whoever manufactured the robot to continuously monitor its user’s collective behavior. Alternatively, they could program the robot to adapt to its user’s behavior over time, perhaps by constantly updating its norm catalogue via community feedback (Malle et al., 2020). However, this approach may face other challenges.

When someone transgresses a norm, potentially indicating that their community will soon abandon it, we cannot immediately tell whether this person has behaved inappropriately or done something that their peers will eventually endorse. Suppose some community members begin ignoring norm X. This will, for all intents and purposes, amount to a transgression. However, these people may prove to be norm entrepreneurs. We cannot know this until their peers collectively begin endorsing their behavior – which they previously would have interpreted as transgressive.

A robot that updates its norm catalogue may have trouble deducing whether it should or should not follow such potential norm entrepreneur’s lead. On the one hand, it may react too slowly when its users begin abandoning a norm. For instance, it may fail to register that it should stop observing the norm as it has interpreted a norm entrepreneur’s actions as transgressions. If this happened, the robot could create the impression that a community endorses a norm that they will soon collectively abandon, potentially slowing down this process by communicating to people that they should continue behaving this way. On the other hand, it may react too quickly. For instance, it may misread someone’s willingness to transgress a norm as an action that signals that their community will soon abandon this way of doing things; and then update its norm catalogue in response. If this community ultimately does not stop observing this norm, they will interpret this robot’s modified behavior as transgressive.

The issue of outdated norms is potentially problematic for some of the strategies researchers have developed to achieve norm-aligned behavior in artificial agents. One of these techniques researchers have used is reinforcement learning (Chen et al., 2017). Particularly, researchers have proposed so-called normative-alignment reinforcement learning to train artificial agents to design robots that adhere to social norms (Nahian et al., 2021). In normative-alignment reinforcement learning, robot designers use a model that biases the re-enforcement learning of an artificial agent towards norm-conforming behavior. Such a model is called *normative prior*. A normative prior model can be trained with examples of normative and non-normative behavior. For instance, a corpus of norm-aligned text, like children’s stories (Nahian et al., 2020). To tune the behavior of an artificial agent to make it norm-aligned with society, the normative prior model can be trained with a corpus that exemplifies the norms of society. However, training with such a corpus of norm-aligned texts could be problematic because the corpus may could include outdated norms, or norms on the verge of becoming obsolete. For instance, children’s stories from 20 or even 10 years may reflect some parenting norms that are out of fashion today.

The danger is that when robots cannot keep up with norm change and is unable to discard outdated norms quickly, they may perpetuate to recently obsolete norms. For instance, a robot may perpetuate harmful norms concerning gender, race, and age. Moreover, in its adherence to outdated norms, the robot may even hinder norm change that the community deems progressive. For instance, by discouraging dissenters or making a norm appear more stable than it is.

### Robot-Induced Norm Change

In earlier sections, we indicated that norm-compliant robots may contribute to the continued observation of norms that their users do not endorse (pluralistic ignorance, outdated norms). In this section, we will examine how robots may contribute to the creation of new norms that undermine practices that individuals and communities value. It is generally accepted within philosophy and ethics of technology that technological innovation almost always produces unforeseeable social consequences (Collingridge, 1980, p.13–23. van de Poel 2016), including the emergence of new norms (Swierstra et al., 2009). We often develop norms to deal with the new ways of doing things made possible by novel technologies. When this happens, we may abandon norms we once followed in favor of new ones centered around a technology’s usage. We often do not endorse such changes and sometimes wish to preserve an older way of doing things that technological innovation has disrupted (Swierstra, 2015). We contend that norm-compliant robots may produce such outcomes and prompt their users to develop and accept new norms that do not benefit them.

Numerous scholars have argued that interacting with social robots can result in the emergence of new practices that conflict with valuable pre-established ways of doing things (Sparrow & Sparrow, 2006; Calo 2010; Dobrosovestnova & Hannibal, 2021). For instance, Sherry Turkle, warns that human-like robots designed for companionship destabilize long-standing norms related to care and affection. Whereas in the past, we relied exclusively on other people to provide emotional support and lend us a sympathetic ear, today, we can delegate such tasks to technologies such as companionship robots or chatbots. Turkle claims that letting these robots serve as stand-ins for human caregivers undermines care practices and norms. She argues that equating simulated interactions fostered by unfeeling, unthinking machines to those we share with people who genuinely care about our well-being cheapens what it means to experience care. Furthermore, this may communicate to (often vulnerable) people suffering from loneliness that they should accept the care provided by robots as good enough, robbing them of the human connections they need to feel that other people do care about them. (Turkle, 2011, p.23–67).

Other scholars have cautioned that norms people rely on to interact with robots may spill over to human interactions (Darling, 2016; Nyholm, 2020, p.27–51, p.181–207). For instance, John Danaher and others, propose that the widespread deployment of sex robots may usher in what can be called a symbolic shift. The argument here is that sex robots can (and often will) represent norms of how one should interact with sexual partner that are ethically problematic and can lead to harmful individual and social consequences (Danaher, 2017). For instance, sex robots cannot feel excited, offended, or nervous and thus do not respond to wanted or unwanted sexual advances as a human would. This sexual deference may encourage some users to treat the robot in a way that is not aligned with norms of consent. Suppose someone mistakenly believes that using a sex robot is a valid representation of the experience of interacting with human sexual partners. In that case, they may fail to respect the norms that communicate mutual consent in a human-human sexual interaction (e.g., all parties involved must voluntarily and enthusiastically agree to proposed sexual relations before they happen).

Other researchers have observed that interacting with technologies that appear to understand human language can alter how one communicates with other people. For instance, several scholars have reported that people who regularly use virtual assistants equipped with voice recognition software can develop speech patterns that sound rude or odd (Wiederhold, 2018; Kudina, 2021). Because these technologies have trouble interpreting anything other than direct commands, long-term users can come to overly rely on this way of speaking and issue imperatives more frequently than considered appropriate during conversations with humans (Wiederhold, 2018; Kudina, 2021). Additionally, these technologies encourage users to omit aspects of speech that they may read as errors, including phrases we use to communicate politeness or friendliness. Indeed, a 2019 study by the British market research firm *YouGov* revealed that more than half of virtual assistant users they surveyed reported they were rude to these technologies (Smith, 2019). One could say that these technologies afford conversational norm transgressions (e.g., the failure to respect that one should not issue too many commands and speak politely), which may contribute to the normalization of such breaches.

These observations and arguments indicate that robots can encourage their users to ignore or fail to learn pre-established norms that govern how one should behave in specific contexts. Considering that the norms discussed above ensure that people receive proper care, treat their sexual partners with respect, and observe conversational etiquette, we have good reasons to claim that they deserve preservation. Abandoning such norms in favor of those that enable us to use a robot may make us worse off and undermine our ability to interact with people as we wish or deserve.

So far, we have chiefly discussed robots that were not explicitly designed to observe norms. If someone programmed these technologies to respect norms that their users and society writ large considers valuable, then, surely, they would help preserve such norms rather than facilitate their abandonment? Although this view seems logical, we will now argue that we should expect norm-compliant robots to encourage potentially unwelcome norm shifts. We will evidence this claim by examining how robots that perform social tasks previously completed exclusively by humans change what it means to do such things.

Let us zoom into norm-compliant robots designed for care. One could imagine that an organization creates a robot that observes the norms nurses generally observe to ensure patients receive proper interpersonal care. Indeed, such machines already exist to some degree (Wright, 2023). This robot respects norms that govern nurses’ bedside manner. It behaves as though it understands that it should respect patients’ privacy, touch them only when appropriate, and communicate clearly but amicably with them – norms nurses generally respect when interacting with patients (Li, van Wynseberghe, & Roeser, 2020). Even if this were the case, the robot’s presence and behavior will almost certainly encourage its users to develop norms to ensure they can use it.

Patients and human caregivers would have to adapt to this machines’ capabilities. Although it may behave like a nurse it cannot do many things that they can and must do. Nurses administer medicine, check patients’ vitals, and deal with emergencies. (Contemporary) robots simply do not have the capabilities to attend to such high-risk tasks. Thus, human nurses will continue to perform them. This means that everyone who interacts with this norm-compliant robot must know what it can and cannot do to ensure patients receive proper care. For instance, patients would have to remember that they should not ask this nurse-like robot to increase their dosage of painkillers and, therefore, should instead command it to call a human nurse when they need someone to perform this task or do so themselves. Likewise, nurses would have to learn that they should not leave patients alone with this robot for too long because it cannot attend to their medical needs even though it behaves like someone who can (van Wynsberghe & Li, 2019).

Learning such things would take time and may ultimately result in the emergence of norms that nurses and patients find troubling. Patients may discover that they preferred to communicate exclusively with human nurses if it meant they did not have to constantly bear in mind what they should and should not ask this robot to do. Likewise, nurses may find that reminding forgetful patients who regularly use this robot that they should call them when they need medical attention is more trouble than it is worth and does not save them time at all (van Wynsberghe & Li, 2019). Indeed, many people who use or work alongside this robot may sense that things were better before its introduction.

This discussion draws attention to another issue that deserves recognition. Robots are not humans. Although a nurse-like robot may simulate what it is like to interact with a human nurse with good bedside manner, it cannot be this person and its introduction may undermine norms that govern what care should look like. Although it is norm-compliant, said robot may call into question whether care is something humans should exclusively provide. If we delegated such tasks to robots, we would have to accept the proscription that “it is acceptable to let robots act as caregivers” – a principle that we have good reasons to reject as it may lead to the normalization of care given by unfeeling, unthinking machines.

We contend that robots designed to stand-in for humans in other domains will produce this outcome too. For instance, norm-compliant law-enforcement robots that behave like police officers would problematize norms associated with who (or in this case what) may sanction citizens when they break the law (Calo, 2011). Likewise, norm-compliant robots designed for educational purposes may disrupt what counts as good teaching (e.g., is it something that only qualified, experienced humans should provide? Or something that a machine that can search the internet for answers without knowing what this information means can and should do?) (Sharkey, 2016). Allowing norm-compliant robots to fulfil social roles of this kind may normalize the idea that robots can and should perform such tasks - even though they cannot do many things we expect from the human beings these machines stand in for (e.g., they cannot genuinely care about patients nor understand the value of teaching). Therefore, compelling us to accept norms that prescribe: “one should let a robot do X social task despite its inability to carry out this task as a human would”.

We contend that norm-compliant robots will induce norm changes. Research clearly demonstrates that technological innovation, including robotics, fosters the development and abandonment of norms. These changes often do not amount to a step in the right direction. Indeed, they can undermine norms that we want to preserve. As such, norm-compliant robots can and almost certainly will contribute to the emergence of new norms that may represent a worse way of doing things.

## Conclusion

We aimed to introduce our readers (many of whom we assume work within robot ethics and social robotics) to critical discussions on norm-compliance and demonstrate why we cannot uncritically rely on norms to build ethically-sound robots. We argued that observing a norm does not mean one has acted well. Indeed, in many cases, we have good reasons to claim the opposite. As such, a robot that observes norms may produce outcomes its users, other stakeholders, and society writ large do not endorse. We contend that discussions of this kind need to be more present in the literature on norm-compliment robots and that researchers from this field generally assume that humans and robots should observe norms. We aimed to convince our readers to think otherwise about norms and develop a resource (our “seven troubles with norms”) that other researchers can use to identify potential ethical or political issues raised by norm-compliant robots.

We will now suggest some preliminary mitigation strategies that other researchers could develop to alleviate the issues we identified. First and foremost, we highly recommend that anyone committed to developing norm-compliment robots integrates relevant sociological and political scholarship, some of which we cited in this contribution, into their research. As stated throughout this paper, we did not discover the “seven troubles with norms” we cataloged. Instead, we were the first to apply them to norm-compliant robots. For instance, political theorists have debated how to avoid tyrannies of the majority and paternalism for over two centuries via principles designed to ensure people can enjoy their lives without being unfairly subjugated to other people’s wills and interests. We will not recount these principles here for the sake of brevity. However, we can recommend two contemporary texts that explicitly and implicitly address these issues respectively: namely John Rawls’ *A Theory of Justice (Rawls,*^1999^).^4^ and Catherine D’Ignazio and Lauren F. Klein’s *Data Feminism* (D’Ignazio & Klein, 2020).^5^. Furthermore, we have referenced several useful sociological and philosophical works in this contribution that attempt to develop strategies to help communities abandon flawed norms, most notably Cristina Bicchieri’s *Norms in the Wild* (Bicchieri, 2017) and Geoffrey Brennan, Lina Eriksson, Robert E. Goodin, and Nicholas Southwood’s *Explaining Norms* (Brennan et al., 2013).

Secondly, some of the troubles we identified could be solved or, at the very least, ameliorated via technical means. For instance, one could address some of the challenges raised in the sections on outdated norms and robot-induced norm change by developing robots that update their norm catalogue over time. A possible mitigation strategy for the challenge of outdated norms, and norm change, in general, is to make a robot more sensitive to changes in the social environment. New approaches to norm-aligned robot behavior, like reinforcement learning with normative prior models (Nahian et al., 2021) mentioned in the section on outdated norms, could be adapted to enable the robot to update its norms. One idea here is to continuously train the robot with new material to update the training data with sources that represent the current norms of society.

And lastly; we would like to make clear that many of the issues we have raised cannot be solved solely via technical means. Nonetheless, roboticists could attempt to lessen their effects by incorporating relevant social scientific methods into their research. Adapting participatory and co-design strategies to build norm-compliant robots could help address the challenge of pluralistic ignorance and outdated norms. In participatory and co-design methods, users and stakeholders are involved in technology design (Steen, 2013; Sanders & Stappers, 2008). Developers of norm-compliant robots could adapt these design approaches so that potential users and other stakeholders give input on the norms a robot is supposed to learn. For instance, robot developers could use focus groups and discussions to investigate people’s attitudes about norms (e.g., ask people whether they truly endorse a norm or observe it because they believe their peers expect them to). Researchers could encourage discussions and deliberation about norms to find out which norms are undesired. Additionally, we highly recommend that anyone who wishes to develop norm-compliant robots practice inclusive design (Walsh & Wronsky, 2019; Clarkson et al., 2003) that includes the voices of marginalized populations. Such inclusive co-design strategies would ideally ensure that minority positions about norms are included, which can help to mitigate the issue of tyranny of the majority.

As concluding remark, we would like to add that we do not believe that other researchers should abandon their efforts to develop norm-compliant robots. This is a fascinating and worthwhile endeavor that may well lead to the creation of robots that benefit their users and society writ large. Instead, we aimed to inspire other researcher to think more critically about norms via this contribution to ensure that this comes to pass.

## Footnotes

1 Cristina Bicchieri for instance stresses expectations in her account of norms. According to Bicchieri, a social norm “[…] is a rule of behaviour such that individuals prefer to conform to it on condition that they believe that (a) most people in their reference network conform to it (empirical expectation) and (b) that most people in their reference network believe they ought to conform to it (normative expectation)” (Bicchieri, 2005, p.35).

2 To be precise, a *substantial* portion of the group needs to follow the norm. Geoffrey Brennan and colleagues (2013, p.29) offer this definition: “A normative principle P is a norm within group G if and only if: (i) A significant proportion of G have P-corresponding normative attitudes; and. (ii) A significant proportion of the members of G know that a significant proportion of the members of G have P-corresponding normative attitudes”.

3 Slurs and hate speech are an exception here.

4 Specifically, in Chapter I: Justice as Fairness and Chapter II: The Principle of Justice.

5 Specifically, p.21–73.
